# A review of signal pathway induced by virulent protein CagA of *Helicobacter pylori*

**DOI:** 10.3389/fcimb.2023.1062803

**Published:** 2023-04-14

**Authors:** Haiqiang Wang, Mei Zhao, Fan Shi, Shudan Zheng, Li Xiong, Lihong Zheng

**Affiliations:** ^1^ Department of Internal Medicine, First Affiliated Hospital, Heilongjiang University of Chinese Medicine, Harbin, China; ^2^ Graduate School of Heilongjiang University of Chinese Medicine, Harbin, China; ^3^ Department of Internal Medicine, Fourth Affiliated Hospital, Heilongjiang University of Chinese Medicine, Harbin, China

**Keywords:** *H.pylori* virulence protein CagA, MAPK signaling pathway, PI3K/Akt signaling pathway, NF-κB signaling pathway, JAK-STAT signaling pathway, Wnt/β-catenin signaling pathway, Hippo signaling pathway

## Abstract

Gastric cancer (GC), a common and high-mortality disease, still occupies an important position in current cancer research, and *Helicobacter pylori* (*H. pylori*) infection as its important risk factor has been a hot and challenging research area. Among the numerous pathogenic factors of *H. pylori*, the virulence protein CagA has been widely studied as the only bacterial-derived oncoprotein. It was found that CagA entering into gastric epithelial cells (GECs) can induce the dysregulation of multiple cellular pathways such as MAPK signaling pathway, PI3K/Akt signaling pathway, NF-κB signaling pathway, Wnt/β-catenin signaling pathway, JAK-STAT signaling pathway, Hippo signaling pathway through phosphorylation and non-phosphorylation. These disordered pathways cause pathological changes in morphology, adhesion, polarity, proliferation, movement, and other processes of GECs, which eventually promotes the occurrence of GC. With the deepening of *H. pylori*-related research, the research on CagA-induced abnormal signaling pathway has been updated and deepened to some extent, so the key signaling pathways activated by CagA are used as the main stem to sort out the pathogenesis of CagA in this paper, aiming to provide new strategies for the *H. pylori* infection and treatment of GC in the future.

## 1 Introduction

GC ranks as the fifth most common cancer and the third leading cause of cancer death in the world, so it is still important cancer worldwide today, and lots of previous studies have confirmed that *H. pylori* infection is a major risk factor for the development of GC and is classified as a class I carcinogen by the WHO (Watanabe et al., 1998; Backert and Tegtmeyer, 2012; Plummer et al., 2015). *H. pylori* contains various pathogenic factors such as cytotoxin-associated gene A (CagA), vacuolating cytotoxin A (VacA), neutrophil activating protein (NAP), outer membrane proteins (OpiA, HopQ) (Palframan et al., 2012), which activate various signaling pathways such as ERK/MAPK, PI3K/Akt, NF-κB, Wnt/β-Catenin, JAK-STAT, Hippo, etc. and promote aberrant transcription of downstream pro-inflammatory/anti-inflammatory, carcinogenic/anti-cancer target genes, which is the key mechanism of *H. pylori*-induced progression of chronic gastritis to GC. As the only bacterial-derived oncoprotein, CagA has been widely studied (Ohnishi et al., 2008; Belogolova et al., 2013; Alipour, 2021). Several studies have confirmed that CagA plays an indispensable role in the pathogenesis of *H. pylori* (Franco et al., 2008), so in this paper, the critical signaling pathways induced by CagA are used as the main stem to comb through the relevant studies on the pathogenesis of this protein, which further clarifies the pathogenesis of CagA-positive *H. pylori* and provides new ideas for clinical blockade of *H. pylori* infection and treatment of GC.

## 2 The structural basis of CagA

**Figure 1:**
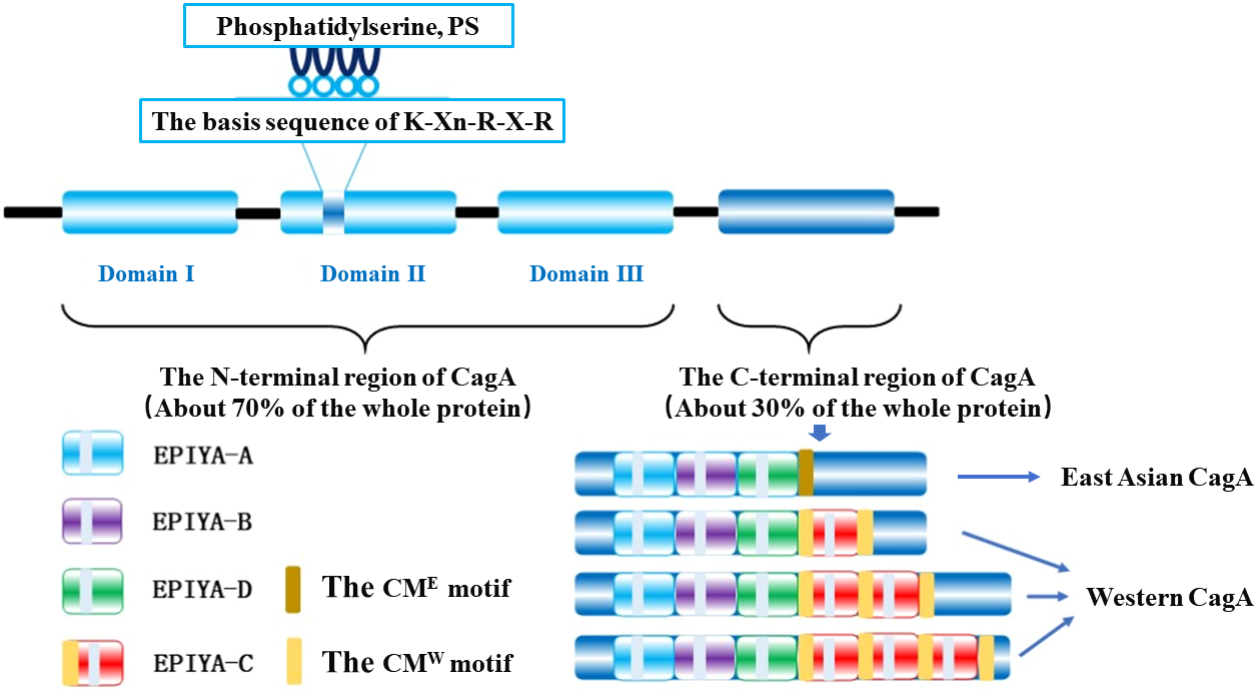
The chematic structure of the *H. pylori* CagA.

CagA is a 128-145 kDa protein (Covacci et al., 1993) that includes a folded N-terminal region (about 70% of the entire protein) and an intrinsically disordered C-terminal region (30% of the entire protein), where the folded N-terminal consists of three different structural domains (structural domains I-III) forming a new protein structure, while the disordered C-terminal region contains a glutamate-proline-isoleucine-tyrosine-alanine motif (Glu-Pro-Ile-Tyr-Ala, EPIYA) fragment and a CagA multimerization motif (CM motif) (**Figure 1**) (Hayashi et al., 2012; Hatakeyama, 2014). There is a small interacting surface region between structural domain I and structural domain II, but no interacting surface region with structural domain III, where the hydrophobic surface N-terminal binding sequence (NBS) of structural domain III interacts intramolecularly with the disordered C-terminal NBS homolog forming a lasso-like disordered loop that together enhances the hub function of CagA pathogenicity (Backert and Tegtmeyer, 2012; Hayashi et al., 2012).

The C-terminal variable region of CagA protein has a repetitive region of the EPIYA Motif, which plays a crucial role in membrane localization in non-polarized host cells and can be classified into four different types according to the amino acid sequence around each EPIYA motif: EPIYA-A, EPIYA-B, EPIYA-C, and EPIYA-D, which constitute the classical EPIYA-repeat region by different combinations and have evident regional variability (**Figure 1**) (Rudi et al., 1998; Higashi et al., 2002; Murata-Kamiya, 2011). The EPIYA repeat region of CagA in Western countries is composed of EPIYA-A/EPIYA-B/EPIYA-C fragments, which are known as Western CagA proteins or ABC-type CagA; while EPIYA-A/EPIYA-B/EPIYA-D fragments, named East Asian CagA proteins or ABD-type CagA, predominate in strains from East Asian countries (Rudi et al., 1998; Higashi et al., 2002; Murata-Kamiya, 2011). Moreover, EPIYA-C fragments are usually present in 1 to 3-fold quantities and in tandem in different Western CagA proteins (Rudi et al., 1998), and only tandem EPIYA-C can activate protein tyrosine phosphatase 2 (SHP2), whereas individually it cannot (Wang et al., 2021). The molecular dynamics simulation of SHP2 confirmed that the binding affinity of EPIYA-D to the N-SH2 domain of SHP2 is more potent than that of EPIYA-C, which explains that part of the reason why the incidence of GC in East Asian countries is much higher than that in western countries is due to the difference of CagA phosphorylation sites on both sides (Hatakeyama, 2004; Wang et al., 2021).

In addition to the EPIYA motif in the C-terminal region, the distal end contains a CM motif consisting of 16 amino acid residues to achieve its motif multimerization (dimerization) upon non-phosphorylation (Saadat et al., 2007); that is, two CagA molecules are recruited through the CM motif (Hatakeyama, 2017) and bind a polarity-regulated kinase 1b (PAR1b) to form the CagA-PAR1b complex, which induces host cell attachment and polarity defects and assists CagA molecules to enter the host cell (Segal et al., 1999; Murata-Kamiya et al., 2010). Although CM motifs are highly conserved in different strains, they are not identical and there is a difference of 5 amino acid changes in CagA of East Asian and Western strains, which are named as CM^E^ sequence and CM^W^ sequence respectively based on this difference. Most East Asian CagA proteins have only one CM sequence downstream of the EPIYA-D fragment. In contrast, in Western CagA, the CM sequence is located in the N-terminal part of the EPIYA-C fragment and downstream of the final EPIYA-C fragment, so the number of CM motifs in Western CagA increases along with the multiplication of the EPIYA-C fragment (**Figure 1**) (Ren et al., 2006; Saadat et al., 2007).

## 3 The translocation and activation of CagA

Upon attachment of *H. pylori* to GECs, CagA enters the host cell through the synergistic action of various molecules such as the type IV secretion system encoded by the Cag pathogenicity island (Cag PAI), externalized phosphatidylserine (PS), multiple adhesion factors (BabA, SabA, OpiA, HopQ, and other outer membrane proteins) and the host cell integrin ɑ5β1 (Backert et al., 2011; Palrasu et al., 2020). Once *H. pylori* enters GECs, CagA is anchored to the inner leaflet of the plasma membrane by different mechanisms depending on the polarity of the cell. In polarized host cells, the interaction of basic amino acids of the Lys-Xn-Arg-X-Arg (K-X-R-X-R) motif with PS plays a major role in the membrane binding of CagA, whereas in non-polarized cells, it is the EPIYA motif that is critical for CagA membrane binding (Hatakeyama, 2014; Hatakeyama, 2017). In addition, *H. pylori* can shed Outer Membrane Vesicles (OMVs) from its outer membrane surface and enter human gastric adenocarcinoma (AGS) cells by macropinocytosis/phagocytosis, like other Gram-negative bacilli (Chew et al., 2021). The OMVs of *H. pylori* were shown to contain adhesins, lipopolysaccharides, and virulence factors (CagA, VacA, and UreA) (Mullaney et al., 2009; Olofsson et al., 2010; Turner et al., 2018), so OMVs are another way for CagA to enter the host cells instead of a functional type IV secretion system (Chew et al., 2021). Studies suggest that CagA is located only on the surface of OMV isolated from *H. pylori* and occurs phosphorylated upon entry into AGS cells, which interferes with cellular signaling pathways leading to inflammation and carcinogenesis (Jarzab et al., 2020), although it is not present in high levels in infected cells (Chew et al., 2021).

CagA immobilized at the plasma membrane undergoes selective tyrosine phosphorylation at the EPIYA site mediated by src family kinase (SFK) and c-Abl tyrosine kinase, where EPIYA-C or EPIYA-D is phosphorylated by SFK at the onset of infection (0.5-2h), followed by rapid inactivation of SFK by phosphorylated CagA and C-terminal Src kinase (Csk) (Tegtmeyer and Backert, 2011), and post-infection (2-8h) c-Abl phosphorylates EPIYA-A or EPIYA-B (Rudi et al., 1998; Tammer et al., 2007; Mueller et al., 2012). So, the phosphorylation process of CagA is not only time-dependent but also kinase selective for motifs. Scholars have also found that the c-Src kinase phosphorylated only EPIYA-C and EPIYA-D, while c-Abl kinase can phosphorylate all repetitive fragments of CagA (Tammer et al., 2007; Mueller et al., 2012). In Western strains, the kinase can preferentially phosphorylate EPIYA-A and EPIYA-C motifs across two CagA molecules or on one CagA molecule at the same time, and it was found that phosphorylation of CagA mostly occurs through one or two EPIYA repeat fragments and rarely three motifs simultaneously (Mueller et al., 2012). Certainly, the phosphorylation of one EPIYA motif alone is not sufficient to induce scattering and elongation in GECs (Takahashi-Kanemitsu et al., 2020). Phosphorylated CagA interacts with SHP2, Csk, Crk junction protein, and other proteins to trigger the ERK/MAPK signaling pathway, leading to abnormal expression of epithelial genes and inducing morphological changes in the “hummingbird phenotype” (Saadat et al., 2007). However, not all pathological changes caused by CagA require the prerequisite of phosphorylation. The CM motif can bind to PARIb, Ecadherin/β-catenin, C-met, growth factor receptor-bound protein 2 (Grb2), or other proteins to disrupt intracellular signaling pathways such as PI3K/Akt signaling pathway and Wnt/βcatenin signaling pathway (Palrasu et al., 2020), so non-phosphorylation plays an equally important role as phosphorylation in the pathogenesis of CagA.

## 4 Signaling pathways activated by CagA

### 4.1 The MAPK signaling pathway

MAPK signaling pathway is a cellular pathway that regulates cell growth, differentiation, stress, inflammation, immunity, and other important physiopathological responses through sequential activation of MAP kinase (MAPK), MAPK kinase (MEK, MKK or MAPK kinase) and MEK kinase (MEKK, MKKK or MAPK kinase kinase) (Raman et al., 2007; Cargnello and Roux, 2011). Among the four major branches of ERK, JNK, p38 MAPK, and ERK5, CagA is dominated by the activation of ERK and JNK subgroups, among which ERK is in charge of cell growth and differentiation, and its upstream signal is the well-known Ras/Raf protein, while JNK is mostly involved in cell inflammation and apoptosis, both of which are critical for CagA-induced GC progression (Cargnello and Roux, 2011).

#### 4.1.1 The MAPK classical signaling pathway

**Figure 2:**
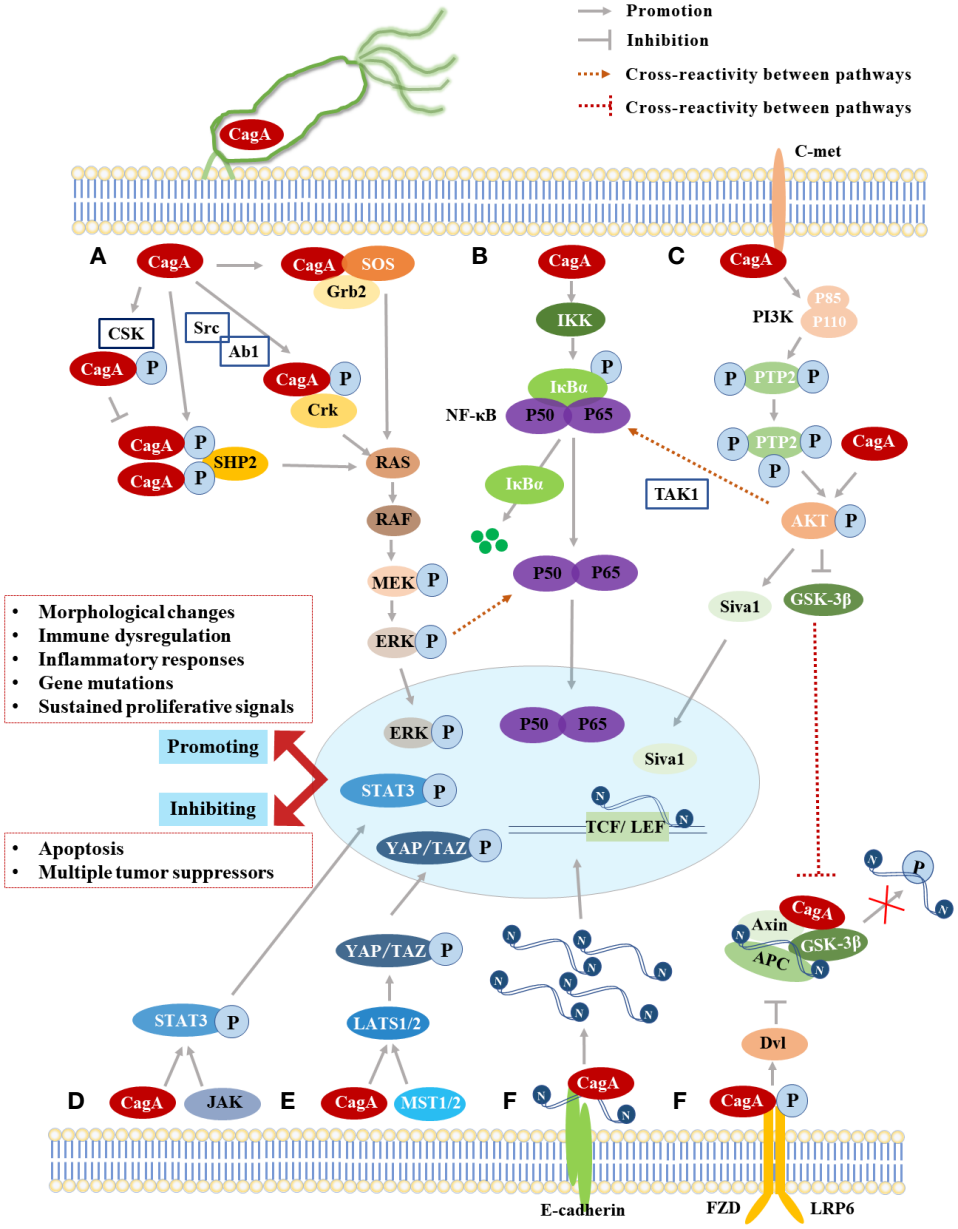
The Summary of CagA-induced signaling pathways. **(A)** The MAPK classical signaling pathway; **(B)** The NF-κB signaling pathway; **(C)** The PI3K/Akt signaling pathway; **(D)** The JAK-STAT signaling pathway; **(E)** The Hippo signaling pathway; **(F)** The Wnt/β-catenin signaling pathway.

CagA acts as a scaffold to recruit SHP2 at the plasma membrane of host cells in a Tyr site tyrosine phosphorylation-dependent manner (Tartaglia et al., 2001). SHP2 is the first known phosphatase to act as a human oncoprotein, which contains two tandem Src homologous structural domains (N-terminal Src-homology domain 2, N-SH2; C-terminal Src-homology domain 2, C-SH2) also is a major molecule in determining the virulence of CagA-positive *H. pylori* (Hatakeyama, 2004; Murata-Kamiya, 2011). CagA can bind to a single SHP2 through two structural domains, N-SH2 and C-SH2, to form a CagA-SHP2 complex, either in cis or trans (Hatakeyama, 2004). Activated SHP2 stimulates ERK through both RAS-dependent and non-dependent pathways, which in turn activates the Ras/Raf/MEK/ERK signaling pathway, thereby deregulating cell proliferation, triggering an abnormal mitotic response, inducing cell elongation to form needle-like protrusions that constitute the morphological changes of the “hummingbird phenotype” (**Figure 2A**) (Higashi et al., 2004; Tsutsumi et al., 2006; Liu et al., 2012; Hatakeyama, 2014). The phenotype is characterized by increased cell viability and scattered elongated cell shape, similar to changes in cell scattering, elongation and diffusion induced by hepatocyte growth factor (HGF) (Segal et al., 1999). It was shown that SHP2 not only participated in the positive regulation of ERK/MAPK activity by CagA but also prolonged the duration of ERK activation, and the morphogenetic activity of CagA was dependent on ERK/MAPK activity, so tyrosine phosphorylation of CagA in this process was necessary to induce the hummingbird phenotype and cell scattering phenotype in GECs (Higashi et al., 2004). Moreover, the CagA-SHP2 complex induces dephosphorylation of various phosphorylation sites of focal adhesion kinase (FAK), an important tyrosine kinase, that controls cell adhesion, spreading, differentiation, motility, and death (Mitra et al., 2005; Tsutsumi et al., 2006), thereby affecting the host cytoskeleton, local adhesion sites bound by extracellular matrix molecules, and structural changes in membrane protrusions through different molecular binding (Tegtmeyer et al., 2017), resulting in alterations in cell motility and cell morphology. The interaction between CagA and PAR1b inhibits the activity of FAK resulting in junction defects and polarity defects in host cells, which promotes the formation of CagA-SHP2 complexes and further induces hummingbird phenotypes (Saadat et al., 2007). Some recent studies have found that CagA can downregulate downstream tumor suppressors genes such as GKN1 and Runx3 after triggering this pathway, thus inducing a decrease in suppressor activity in GECs and ultimately promoting the development of GC (Tsang et al., 2010; Guo et al., 2020).

The phase interaction of CagA-SHP2 was mediated by the phosphorylation of EPIYA-C or EPIYA-D fragments, while the phosphorylation of EPIYA-A or EPIYA-B fragments participates in the interaction of CagA-Csk, which is similar to the complex formed after SHP2 activates Csk (Murata-Kamiya, 2011). The CagA-Csk formation competitively inhibits CagA-SHP2 binding and down-regulates CagA-SHP2 signaling by decreasing the phosphorylation level of CagA (**Figure 2A**) (Tsutsumi et al., 2003). Therefore, the interaction of CagA with Csk may establish a negative feedback regulatory loop to prevent excessive cell damage caused by excessive activation of phosphorylation-dependent CagA activity (Hatakeyama, 2014), which is conducive to persistent infection of CagA-positive *H. pylori* while avoiding excessive toxicity to host cells, so Csk is considered as a negative regulator of SFK (Nada et al., 1991; Tsutsumi et al., 2003). The inhibition of SFK kinase activity by the CagA-Csk complex also affects the phosphorylation status of actin-binding proteins (cortactin, vinculin, etc.) (Hatakeyama, 2014), leading to an overall rearrangement of the actin cytoskeleton, which promotes cell motility, scattering, and elongation (Tegtmeyer and Backert, 2011), further contributing to the “hummingbird phenotype” of cell morphology changes. In addition, phosphorylated CagA interacts with Crk junction proteins (Crk-I, Crk-II, Crk-L) to induce other downstream signaling pathways such as SoS1/H-Ras-Raf-MEK and C3G-Rap1/B-Raf-MEK (Suzuki et al., 2005), which plays an important role in promoting cell scattering (Chen et al., 2016). In the non-phosphorylated state, the CM motif of CagA interacts with Grb2 to activate the RAS/MEK/ERK pathway and lead to cell dispersion and proliferation, but the EPIYA fragments required for tyrosine phosphorylation are indispensable in Grb2 binding and cellular response (**Figure 2A**) (Mimuro et al., 2002).

The ERK/MAPK signaling pathway is the core of the signaling network that regulates cell growth, development, and division (Tang et al., 2017), and timely blockage of the pathway may be a potential way to prevent GC. Berberine inhibits the proliferation and tumorigenicity of GECs by inactivating the MAPK signaling pathway, and reduces the secretion of IL-8, thus playing a good role in the treatment of gastric cancer (Li et al., 2016).

#### 4.1.2 The JNK signaling pathway

The JNK signaling pathway is an important branch of the MAPK signaling pathway that plays an important role in a variety of physiological and pathological processes, including cell cycle, reproduction, apoptosis, and cellular stress (Gozdecka et al., 2014). It has been proved that the JNK signaling pathway cannot only inhibit tumors but also promote tumors in different cell types and organs (Epstein Shochet et al., 2014). Through the experiments of transgenic fruit flies, some scholars have found that CagA can trigger the activation of the JNK signal pathway and induce apoptosis of epithelial cells (Wandler and Guillemin, 2012). JNK-mediated apoptosis may play a role in limiting pathogenicity and protecting GECs during early infection, but the accumulation of genetic mutations may occur under the combined influence of persistent *H. pylori* infection and other factors, which will promote tumor progression when carcinogenic mutations are acquired (Yong et al., 2015).

The new study also suggests that early in *H. pylori* infection, phosphorylated CagA promotes cortical actin overexpression through activation of JNK and stimulates actin-cytoskeleton, cell adhesion, and motility changes, which affect cell structure and epithelial barrier function in the development of GC (Sharafutdinov et al., 2021).

### 4.2 The PI3K/Akt signaling pathway

The PI3K/Akt signaling pathway is mainly involved in protein synthesis, cell survival, migration, and growth (Franke, 2008). The activation of the pathway is in response to growth factors, such as epidermal growth factor and hepatocyte growth factor (Yong et al., 2015). The classical cascade reaction is to activate the heterodimer (Phosphatidylinositol-3-kinase, PI3K), which is composed of the p85 regulatory subunit and p110 catalytic subunit, through specific receptors (EGFR, C-met) and other molecules (Franke, 2008; Yong et al., 2015). The activated PI3K can phosphorylate the second messenger phosphatidylinositol (3,4)-biphosphate (PIP2) to generate phosphatidylinositol (3,4,5)-trisphosphate (PIP3) and cause Akt kinase phosphorylation at the Ser473 or Thr308 sites, which in turn activates or inhibits downstream targets such as glycogen synthase kinase 3β (GSK-3β) and forkhead box protein o (FOXO), and this pathway signaling was found to be active in tumor cells such as GC (Franke, 2008; Saijilafu et al., 2013; Yong et al., 2015). Under the regulation of the PI3K/Akt pathway, CagA induces the phosphorylation of X-linked inhibitor of apoptosis protein (XIAP) E3 ubiquitin ligase at position 87 and initiates ubiquitination and proteasome dissociation of the pro-apoptotic factor Siva1, which leads to the inhibition of apoptosis and DNA damage response (**Figure 2C**) (Palrasu et al., 2020). CagA also promotes GC by mediating autophagy through the C-met/Akt signaling pathway and promoting the expression of downstream inflammatory cytokines (Li et al., 2017a).

The PI3K/Akt signaling pathway is essential in the pathogenesis of CagA, which can be activated through the following three pathways: (1) When CagA is not phosphorylated, the CM motif interacts with the hepatocyte growth factor receptor-C-met (Suzuki et al., 2009), representing activation of PI3K/Akt signaling *via* a phospholipase Cγ (PLCγ)-related junction protein (Chen et al., 2016), causing inactivation of the downstream target gene GSK-3β and subsequently inducing crosstalk of the Wnt/β-catenin signaling pathway and NF-κB signaling pathway to promote the cell proliferation and enhance the inflammatory response (Suzuki et al., 2009; Tabassam et al., 2009). (2) CagA interacts with PI3K *via* the tyrosine phosphorylation motif (B-TPM) of EPIYA-B repeat region, thereby inducing the PI3K/Akt signaling pathway (Yong et al., 2015; Zhang et al., 2015). The B-TPM has the A/T polymorphism of EPIYA and EPIYT and has an apparent non-random geographical distribution, with EPIYT B-TPM being more predominant in Western *H. pylori* isolates, and structural modeling revealed that this is due to the side chain hydrogen bond formed by the threonine residue at the pY^+1^ position with PI3-kinase N-417 increasing its affinity for PI3K binding (Yong et al., 2015; Zhang et al., 2015). However, EPIYA is more dominant in East Asian *H. pylori* isolates, does not have this affinity (Matsunari et al., 2012). So CagA regulates the interaction with PI3K through the A/T polymorphism of B-TMP, which regulates the activity of the oncogenic-related PI3K/Akt signaling pathway and enhances the risk of GC (Huang et al., 2008; Yong et al., 2015; Zhang et al., 2015). (3) Scholars have found that CagA can phosphorylate Akt 1 (Protein Kinase B, PKB) and activate the ubiquitin ligase Hdm2 to induce degradation of the tumor suppressor p53 in GECs from *H. pylori*-infected Mongolian gerbils (Wei et al., 2010), which is triggered by the direct interaction of ectopically expressed CagA with AKT (**Figure 2C**) (Tegtmeyer et al., 2017). The PI3K/Akt signaling pathway contributes to the progression of GC through epithelial-mesenchymal transition (EMT) stimulation, and resveratrol was able to inhibit Doxorubicin treatment EMT-mediated resistance by significantly reducing the Akt signaling pathway (Kiu and Nicholson, 2012). So, the PI3K/AKt may also serve as a target for the treatment of *H. pylori* infection.

### 4.3 The NF-κB signaling pathway and JAK-STAT signaling pathway

#### 4.3.1 The NF-κB signaling pathway

Downstream of the PI3K/Akt signal pathway, NF-κB is an important inflammatory target, which acts as an essential nuclear transcription factor regulating the inflammatory response/immune response of bodies and apoptotic, stress response of cells (Yong et al., 2015). NF-κB is a heterodimer composed of p65/p50 and forms a complex with IκBα, the intracellular inhibitory proteins, in the classical pathway, and the phosphorylation of the p65 subunit in NF-κB plays a crucial role in its own transnuclear process (Yong et al., 2015). The resting IκB kinase (IKK) is activated by some extracellular signals mediated by membrane receptors, and then phosphorylates, ubiquitinates and detaches IκBα protein from the complex, which is eventually cleaved by proteasomes and releases NF-κB dimer (Neumann and Naumann, 2007). The activated NF-κB is transferred to the nucleus of cells and binds to specific sequences of DNA to further promote the transcription of the target gene (Neumann and Naumann, 2007).

The PI3K/Akt signal pathway usually promotes the degradation of IκBα protein and the nuclear transfer of NF-κB through the order of CagA-C-met-PI3K-AKt axis (Takahashi-Kanemitsu et al., 2020), and ubiquitination of transforming growth factor-activated kinase 1 (TAK1) plays a crucial role in this process (**Figure 2B**) (Lamb et al., 2009). CagA binds to TAK1 in the pathway and mediates Lys 63-linked TAK1 ubiquitination *via* tumor necrosis factor receptor-associated molecule 6 (TRAF6) (Wang et al., 2001). The activated TAK1 induces phosphorylation of the IKK complex, which ultimately activates the NF-κB signaling pathway (Lamb et al., 2009), releases the inflammatory cytokine interleukin-8 (IL-8) (Brandt et al., 2005), and induces phospholipase D1 (PLD1) expression (Kang et al., 2013). Investigators also found that the phosphorylated EPIYA-C motif of CagA also induced the activation of NF-κB and up-regulated IL-8, but it was not related to the number of repetitions of EPIYA-C fragments (Papadakos et al., 2013). The activation of NF-κB and the release of IL-8 were also found to be related to the activation of the ERK signaling pathway by CagA, but the effect is modest (Kim et al., 2006). This pathway mediates the direct binding of NF-κB to the Rev-erbα promoter, thereby increasing bacterial colonization within the gastric mucosa (Mao et al., 2021). CagA-activated NF-κB can induce aberrant expression of cytidine deaminase (AID), a key gene for antibody gene diversification in GECs, resulting in a high mutation frequency of the tumor suppressor p53 (Matsumoto et al., 2007). NF-κB also directly binds to the promoter of miR-223-3p in a CagA-dependent manner and stimulates the up-regulation of miR-223-3p, which induces ARID1A gene expression to decrease and promotes the proliferation and migration of GC cells (Yang et al., 2018). Therefore, it is well documented that CagA-activated NF-κB plays an important role in the development of GC. Inhibiting NF-κB can modulate the expression of inflammatory factors to alter the microenvironment, which can also regulate the expression of oncogenes to slow down the progression of GC, and berberine can treat stomach cancer by this (Liu et al., 2022).

#### 4.3.2 The JAK-STAT signaling pathway

The JAK-STAT signaling pathway consists of three components: tyrosine kinase-associated receptors that receive the signal, tyrosine kinase JAK that transmits the signal, and transcription factor STAT that produces the effect (Yong et al., 2015). As a stress inflammatory signal pathway, this pathway is an effective mechanism to limit the pathogenic effect and protect GECs in early CagA infection (Higashi et al., 2004). However, when CagA excess activates NF-κB and/or STAT3, it could induce the production of pro-inflammatory cytokines and anti-apoptotic proteins to promote the expansion of cancer-susceptible cells and prevent their apoptosis, which also induces ROS to increase DNA damage and accelerate the accumulation of mutations (Hatakeyama, 2014; Yong et al., 2015). When carcinogenic mutations are obtained, JAK-STAT signaling pathways are activated and accelerate the progression of a variety of tumors, including GC (**Figure 2D**).

After infection with CagA-positive *H. pylori*, the upregulation of IL-6 and IL-11 expression in GECs depends on non-phosphorylated CagA, and induces STAT3 tyrosine phosphorylation through the gp130 subunit of the IL-6 receptor to manipulate host immunity and promote immune evasion (Jackson et al., 2007; Rizzuti et al., 2015; Hatakeyama, 2017). The gp130 subunit plays a central role in regulating the balance between SHP2/ERK signaling and JAK-STAT3 signaling, and the phosphorylation status of CagA further affects GC progression by affecting the signaling switch between the SHP2/ERK and JAK/STAT3 pathways *via* gp130 (Lee et al., 2010). It was shown that the STAT3 signaling pathway activated by CagA increases the expression of the bactericidal agglutinin REG3γ (Lee et al., 2012), and CagA can also regulate the secretion of IL-10 and the phosphorylation of STAT3 to damage the function of dendritic cells and manipulate T cell immune response, which is conducive to persistent *H. pylori* infection and increases the probability of GC (Kaebisch et al., 2014). Immune drugs are widely used in GC treatment, which inhibit JAK-STAT signaling can achieve both targeted blockade and avoid creating an immunosuppressive environment, so JAK and START inhibitors have good application prospects in cancer research, and combined immunosuppressants provide new therapeutic ideas for GC patients (Kiu and Nicholson, 2012; Li et al., 2016).

### 4.4 The Wnt/β-catenin signaling pathway

The Wnt/β-catenin signaling pathway is a class of highly conserved signaling pathways during species evolution, which plays a critical role in early embryonic development, organogenesis, tissue regeneration, and other physiological processes in animal embryos (Hatakeyama, 2014; Yong et al., 2015). The core of this pathway is the multi-functional protein β-catenin encoded by CTNNB1 (Hatakeyama, 2014). Under physiological conditions, β-catenin interacts with the cytoplasmic tail of E-cadherin to form adhesion junctions between epithelial cells, and if this protein is affected, it is likely to trigger abnormal activation of the pathway and thus induce cancer development (Clevers, 2006). When GECs are infected by CagA-positive *H. pylori*, CagA and E-cadherin become competitive binding proteins, which destroys the formation of the complex between E-cadherin and β-catenin, leading to the accumulation of β-catenin in the cytoplasm and nucleus and subsequently triggering the Wnt/β-catenin signaling pathway, which requires the EPIYA repeat region of CagA but does not depend on CagA’s own tyrosine phosphorylation (**Figure 2F**) (Kurashima et al., 2008; Yong et al., 2015).

The classical Wnt/β-catenin signaling pathway is triggered by the binding of extracellular Wnt ligands to the transmembrane receptor Frizzled family protein (FZD) and its accessory receptor low-density lipoprotein receptor-related protein (LRP) (Kimelman and Xu, 2006). Activated FZD receptors initiate intracellular signal cascades and phosphorylated LRP activates Dvl proteins, which together phosphorylate the N-terminal end of β-catenin through the destruction complex (consisting of GSK3β, core protein (Axin), colon cancer-associated oncogene (APC), and casein kinase-1 (CK-1)), and the phosphorylated β-catenin is ubiquitinated by β-transducin repeats-containing proteins (β-TrCP) and covalently modified by the intracellular proteasome, which keeps the amount of intracytoplasmic β-catenin at a low level (Kimelman and Xu, 2006; Klaus and Birchmeier, 2008). However, when GECs were infected with CagA-positive *H. pylori*, Wnt signaling leads to the inactivation of the destruction complex and thus fails to phosphorylate intracellular β-catenin, causing a large accumulation of β-catenin in the cytoplasm and entering the nucleus to interact with T-cell factor/lymphatic enhancer factor (TCF/LEF) family transcription factors, which induces the expression of cancer-related genes such as Cyclin D1 and c-myc to affect cell differentiation, proliferation, migration, and adhesion, leading to tumorigenesis (**Figure 2F**) (Franco et al., 2005; Klaus and Birchmeier, 2008; White et al., 2012). *H. pylori* can activate the Wnt/β-catenin signaling pathway in GC cells, which enhances cell invasion and angiogenesis. Inhibiting the activation of this pathway can achieve anti-tumor effects, such as Wnt/β-catenin signaling pathway inhibitors and berberine, which can play a similar role in regulating GC cells (Liu et al., 2022), so the Wnt/β-catenin signaling pathway has the potential to become an effective way to prevent and treat GC.

### 4.5 The Hippo signaling pathway

The Hippo signaling pathway, as a conserved pathway originally defined through Drosophila protein kinase (Hippo), mostly controls organ size by regulating cell proliferation and apoptosis, and acts as a key role in embryonic development, organ growth, tissue regeneration, stem cell pluripotency, and tumorigenesis (Chen et al., 2019). There is no doubt that Hippo signaling pathway also plays a crucial role in the development of GC (Qiao et al., 2018). The pathways include Sav1 protein, MOB1, MST1/2, LATS1/2, and two downstream effectors containing WW domain (YAP and TAZ) (Salah and Aqeilan, 2011; Piccolo et al., 2014). YAP is a core component of the Hippo pathway and its increased expression is closely associated with different human tumor progression. When receiving conventional signals, MST1/2 phosphorylates the Thr1079/Thr1041 site of LATS1/2 upon stimulation by SAV1 and MOB1, and activated LATS1 directly phosphorylates YAP, which generates cytoplasmic isolation from the nucleus by binding to 14-3-3 proteins, ultimately limiting tissue overgrowth (Moroishi et al., 2015; Denic et al., 2020). However, when the signaling pathway is closed, the activated YAP enters the nucleus in the presence of transcription factors TEADs to induce the expression of oncoproteins such as connective tissue growth factor (CTGF) and cysteine-rich angiogenic inducer 61 (CYR61), which further promoting cancer development (Moroishi et al., 2015; Li et al., 2017b; Li et al., 2018).

Several studies have demonstrated that CagA-positive *H. pylori* infection induces abnormal Hippo signaling in GECs and promotes the activation and nucleation of the critical effector molecule YAP, during which CagA upregulates LATS2 and significantly increases the expression of YAP and TAZ, which induces the expression of downstream target genes, thereby inducing EMT and intestinal epithelial to increase the risk of early GC (**Figure 2E**) (Mo et al., 2015; Molina-Castro et al., 2020). Studies have shown that the activation of YAP into the nucleus increases in *H. pylori*-positive chronic gastritis, the expression of E-cadherin decreases and the expression of CTGF and CYR61 increases, which ultimately leads to the invasion and migration of gastric adenocarcinoma cells and promotes oncogenic EMT transformation (Li et al., 2018). Blocking the abnormal activation of the Hippo signaling pathway through inhibiting YAP is also one of the ways to treat GC (Xiao et al., 2023).

## 5 Conclusions and perspectives

GC is one type of cancer that develops gradually through a multi-step histopathological cascade reaction over a long period of time (Hatakeyama, 2014). Breaking through its treatment bottleneck is still one of the challenges in the medical field today, and *H. pylori* infection as a key factor inducing GC is naturally a key breakthrough point. CagA is undoubtedly the most critical entry point as the best of many pathogenic factors of *H. pylori*. It is not difficult to find that disrupting key cell signaling pathways and inducing various pathological changes in host cells is the key to the oncogenicity of CagA by synthesizing the exposition in this paper. Abnormally transduced signaling pathways induce the following changes in the host, among others: (1) Promoting morphological changes (Hummingbird phenotype, EMT); (2) Facilitating immune dysregulation and exacerbating inflammatory responses; (3) Inducing gene mutations and inhibiting apoptosis, releasing sustained proliferative signals to increase cancer risk; (4) Regulate gene transcription, downregulate multiple tumor suppressors such as RUNX3, p53, and Siva1, and reduce oncogenic activity. These findings systematically demonstrate the mechanism of CagA in *H. pylori*-induced transformation of chronic gastritis to GC, so blocking the expression of key factors in MAPK signaling pathway, PI3K/Akt signaling pathway, NF-κB signaling pathway, JAK-STAT signaling pathway, Wnt/β-catenin signaling pathway, and Hippo signaling pathway, will be a new strategy to eradicate *H. pylori* and treat GC.

## Author contributions

HW, MZ, and LZ concepted and designed the review. MZ, FS and SZ wrote the manuscript. HW, LX, and LZ revised the manuscript. HW and MZ should be considered joint first author. All authors contributed to the article and approved the submitted version.
